# Additive Manufacturing of Thermoplastic Polyurethane-Cork Composites for Material Extrusion Technologies

**DOI:** 10.3390/polym15153291

**Published:** 2023-08-03

**Authors:** Mario Alvarez Gómez, Daniel Moreno Nieto, Daniel Moreno Sánchez, Alberto Sanz de León, Sergio Molina Rubio

**Affiliations:** 1 Departamento de Ingeniería Mecánica y Diseño Industrial, Escuela Superior de Ingeniería, IMEYMAT, Campus Río San Pedro, Universidad de Cádiz, Puerto Real, 11510 Cádiz, Spain; mario.alvarezgomez@alum.uca.es (M.A.G.); danielmoreno.sanchez@uca.es (D.M.S.); 2 Departamento de Ciencia de los Materiales e Ingeniería Metalúrgica y Química Inorgánica, F. Ciencias, IMEYMAT, Campus Río San Pedro, Universidad de Cádiz, Puerto Real, 11510 Cádiz, Spain; alberto.sanzdeleon@uca.es (A.S.d.L.); sergio.molina@uca.es (S.M.R.)

**Keywords:** additive manufacturing, fused granular fabrication, TPU, cork, composites, mechanical properties

## Abstract

Among the material extrusion technologies of additive manufacturing, fused granular fabrication is playing a bigger role in the industry. The increase in the size of printers demands extrusion systems with higher deposition rates that facilitate printing larger parts in shorter times with a need for cost reduction. This cost reduction in fused granular fabrication systems is due to the utilisation of pellets as the material source for the prints, such as pellets that are the most common way of distributing polymeric materials in industry and do not need the usual previous transformation into filaments. Most of the polymers in the industry can be found in the shape of pellets, so the opportunities for developing new materials beside the traditional filaments found in the market are expanding. In this research, a novel composite material has been developed based on the blending of commercial thermoplastic polyurethane (TPU) and cork particles obtained from industrial waste at different concentrations. These materials have been processed at a laboratory scale, and their mechanical, thermal and rheological properties have been studied. Despite a 53.52% reduction in the maximum stress on the x-axis, an 81.82% decrease in the values obtained with specimens oriented on the z-axis and a shortage in the deformation values, the results reveal a remarkable weight reduction leading to 21.31% when compared to the TPU of the blends,. These results may open a path to further explore these blends and find suitable applications in industry as proposed.

## 1. Introduction

Fused pellet modelling (FPM), polymeric pellet-base additive manufacturing (PPBAM) and fused granular fabrication (FGF) are some of the denominations that have recently been adopted in the literature to refer to a specific additive manufacturing (AM) approach where the material feed source is polymeric pellets [1,2]. This AM approach is among the seven different categories proposed by the International Organization for Standardization (ISO) and American Society for testing materials F42 committee [3], established in a polymeric material extrusion (ME) process.

In this specific process, the source material is directly melted and deposited through an extruder, which allows us to scale up the size of the deposited bead and therefore the entire additive manufacturing process, as nozzle diameters can increase with larger deposition rates that easily achieve several kilograms per hour. This process keeps the original AM approach, whereby a three-dimensional object or part is built layer by layer, depositing material only where it is necessary [4].

The application of these specific extruders allowed the configuration of new specific equipment that has already been applied in the automotive, energy, naval or aeronautical industries [5,6,7], while reducing the environmental impact in depositing the material only where needed. Some of the most important technological developments in this area have been the big area additive manufacturing (BAAM) at Oak Ridge National Labs (ORNL) [8] and the large-scale additive manufacturing (LSAM) by Thermwood [9], generally oriented to what is called large-format additive manufacturing (LFAM) [10], but these can also be applied at different scales. This process is still slow compared with conventional manufacturing, but due to its versatility and flexibility, it can cover specific needs in the industry, such as large prototyping, moulding and tooling, especially when combined with milling.

The novelty of this scaled-up industrial process has also generated the need of specific materials, specifically, polymeric blends that could fulfil the specific requirements of the process. These materials should respond to the demanding requirements of the ME process by providing a specific thermal, rheological or mechanical behaviour to allow its extrusion and prevent deformations, warping, delamination or cracking of the printed parts [11,12]. Another important requirement is the reduction in the carbon footprint. In this sense, the use of FGF systems has boosted the opportunities for developing new materials as it works with pellets, thus providing a greener effective alternative. This not only facilitates the use of compatible materials together with natural additives, but it also decreases their final price, as there is no need to transform the pellets into filaments, which is necessary in the Fused Filament Fabrication (FFF) process. Also, the increase in the nozzle diameter up to several millimetres in size facilitates the use of different additives or larger fibres. This is developed in the context of an overall strategy of reducing the footprint of polymeric materials with the incorporation of bio-residues, thus reducing the use of fossil-based materials.

Thermoplastic polyurethanes (TPUs) are widely used in ME additive manufacturing processes. These materials are composed of a hard segment, formed by adding a chain extender (for instance 1,3-Butadiene-1,4-diol) to an isocyanate functional group, and a soft segment, formed by flexible polyether or polyester chains that connect two hard segments, conferring an elastic behaviour to these materials [13]. TPU has been combined with different additives. For instance, Bi et al. studied the combination of poplar wood particles with TPUs for FFF, analysing the behaviour of different additives, adding 5% to the samples of 20 wt% organic filler [14]. Tan et al. blended TPU, TPU-PEO (Polyolefin Elastomer), TPU–NBR and NBR (Nitrile Butadiene Rubber) with a 20 wt% of corn starch, which resulted in a decrease in the percentage of deformation while maintaining its tensile strength, thus making the material more rigid and decreasing impact resistance, that is, generally decreasing its mechanical properties [15].

Cork is a natural material extracted from a variety of oak trees (*Quercus suber*), and it has been used since ancient times due to its lightweight, watertight and thermal insulation properties. Some common applications are bottle caps, isolation material, floors, sportive equipment or consumer products. Also, the aerospace industry has used it as a thermal shield for landing and take-off as it forms a layer of carbon that acts as an insulator while the layer below it continues to decompose and release gases to cool the surface [16]. It has also been used in bio-filters and as a heavy metal absorber, replacing active carbon or improving impact resistance at low speeds in epoxy resins [17]. Cork has also been used in additive manufacturing as a filler to develop bio-based composites with enhanced mechanical or functional properties. For instance, Daver et al. developed cork–PLA composites for AM and compared the printed material properties with its compression-moulded counterpart [18]. The printed specimens presented a lower Young’s modulus, a slightly lower yield point and slightly higher elongation at break than that those manufactured via compression. Magalhães da Silva et al. prepared two samples of PLA/cork at 15 wt. %, one of them with 4 wt.% of maleic anhydride, and compared injection-moulded specimens with 3D-printed ones [19]. The results reveal a better mechanical behaviour for injection-moulded (IM) specimens with maleic anhydride and a similar behaviour for this composition between IM specimens and those that are additive manufactured. Recently, we also developed a series of cork-based composites for FFF using ASA as a matrix. We showed that the hydrophobization of the cork particles’ surface enhances their compatibility with the thermoplastic matrix, increasing the mechanical properties of the composite [20,21].

The combination of TPU and cork can also provide potential benefits together with specific properties. Using cork as a filler could help to reduce the cost and weight of the materials; residual cork powder has a positive environmental impact. Gama et al. [22] studied the compression behaviour of different foam content of TPU/cork structures printed by FFF, concluding that the properties of these composites depend on the homogeneity of the matrix and filler mixture, the particle size and the cohesion of the mixture. This is, to the best of our knowledge, the only report where TPU/cork composites are used for AM.

Present research attempts to develop new alternatives in the development of TPU composites for FGF. The selected additive for this study is cork powder, a natural material that is frequently disposed of as a subsidiary product from several industries. This could allow the incorporation of industrial waste into the development of new materials together with the exploration of the specific properties of the cork in FGF processes.

More precisely, in this article, the procedure of blending TPU with cork particles is presented, with a detailed definition of the process and problems aroused. Thermal and rheological characterization of the blends was performed to validate its processability and define the temperature parameters range. Then, a number of monolayer specimens at different concentrations were printed in a desktop FGF system. These specimens have been tested and their mechanical behaviour to tensile test presented and discussed.

## 2. Materials and Methods

TPU (Elastollan B95 A 11 000) was provided by BASF (Lemförde, Germany) as pellets. Cork particles with an average density of ρ = 200 kg/m^3^ were supplied by the company Corchos del Estrecho (Cádiz, Spain), obtained from cork dust residues from the sanding processes during the manufacture of cork stoppers. The cork powder was sieved in an electric shifter using different sieve net sizes (i.e., 0.45, 0.154 and 0.025 mm), using a fraction in the range 0.154–0.025 mm. Also, moisture absorption should be taken into account, as usually these natural fillers have higher absorption rates [14], so prior to any use, the materials were dried in a VACUTherm (Thermo Scientific, Germany) for 18 h at 60 °C.

**Figure 1:**
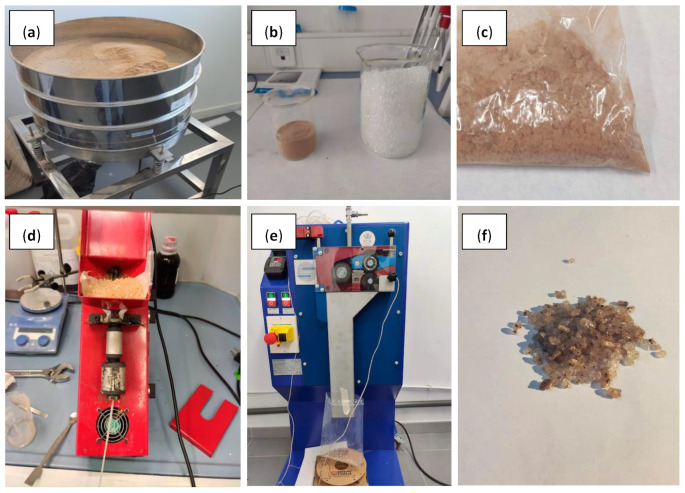
Different stages of the blend process at a laboratory scale: (**a**) cork powder sieved in an electric shifter; (**b**) sieved cork powder and TPU raw material; (**c**) cork powder and TPU after manual mixing; (**d**) filament manufacturing in a single-screw extruder Noztek Pro; (**e**) cutting process of pelletizer Scamex; (**f**) pellets obtained.

TPU and cork powders were manually mixed in a concentration of 1 and 3 wt.%. Blends and neat TPU were processed at 240 °C in a laboratory single screw, Noztek Pro laboratory extruder (Noztek, UK, L/D 26:14 cm, 60 rpm). A continuous filament of each material was produced and cut into small pieces (4–5 mm length) in a pelletizer (Scamex, France); the resulting pellets were used as feedstock in FGF. The chronological procedure is shown in Figure 1.

Monolayer specimens for tensile testing were printed in a desktop printer Creality Ender 3, modified with a vertical Pellet extruder MAHOR V.4 (Mahor·xyz, Andosilla, Spain), with a 0.8 mm nozzle. The extruder system (L/D 8:0.8 mm) has a deposition rate of 0.2 Kg/h, print speed of 60 mm/s, travel speed of 120 mm/s and acceleration of 500 mm/s^2^. The printer is also upgraded with lineal guides in X, Y and Z axis, a motherboard with drivers TMC2209, double Z axis, a hot end of 50 W up to 300 °C and firmware Marlin 2.0.9. Table 1 presents the rest of the printing parameters.

**Figure 2:**
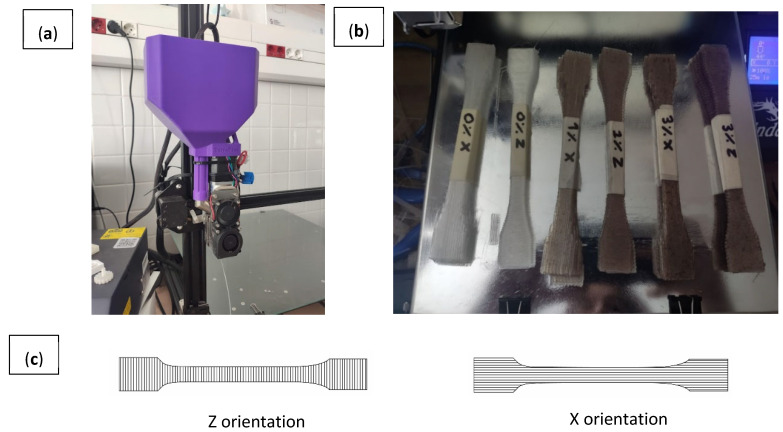
(**a**) FGF system; (**b**) printing configuration of samples; (**c**) samples printed.

Monolayers based on A2 tensile testing specimens (UNE-EN ISO 20753: 2019/ASTM D638) were printed in both the longitudinal and transversal directions located in the XY plane according to the axis definition in the ISO/ASTM 52921-13 standard [23,24], i.e., the X and Y axis. The Y axis simulates the layer adhesion that would exist in the Z axis, placing the filler transversely to the specimen, as it was not possible to print vertically due to the elastic nature of the specimens and the inconsistent flow of the blends. Hence, the samples printed in this way are labelled as “Z” (Figure 2).

The printed specimens were tested in a Shimadzu AGS-10kN tensile testing machine at 50 mm/min following specific considerations for elastomeric materials according to UNE ISO 37:2013/ASTM D638. Five specimens were printed for every configuration with the X and Z orientation for every blend, making a total of 30 specimens. The specimens were modelled with Solidworks™ (Dassault Systems, Paris, France) computer-aided design software with a 0.01 mm deviation tolerance.

Melt flow index determination was performed with the LR-A001-A equipment from Lonroy, heating the samples to temperatures in the range of 210–220 °C and with 5 Kg loads. Differential scanning calorimetry (DSC) and thermogravimetric analysis (TGA) were carried using TA Instruments Q600 simultaneous DSC-TGA (SDT) equipment. For DSC, a first temperature sweep from room temperature to 250 °C was performed to remove the thermal history of the material. Then, a cooling and a heating sweep were performed to obtain the cold crystallization and melting temperatures and the enthalpies. All the sweeps were performed at a rate of 10 °C/min under 50 mL/min nitrogen flow. The temperature sweep for TGA was performed from room temperature to 600 °C at a rate of 10 °C/min under 60 mL/min nitrogen flow.

## 3. Results

Monolayers have been reported as a feasible way to evaluate the intrinsic properties of AM structures. Monolayers are the minimum entity printed that might determine the influence of defects and printing orientation in the resultant properties [25,26]. It is important to highlight that during printing, an increase of 216% in the flow rate was needed to process the blends compared with neat TPU. That increase in the flow rate for blends during printing suggests that the blends have an increase in viscosity, which is reported in previous works [27,28], and justifies, to some extent, the impossibility of processing a higher loading of cork in single screw extruder. As cork has a density of 0.12 g/cm^3^ [29] and a TPU of 1.22 g/cm^3^, a small percentage of filling by mass represents a large percentage by volume, which presents a notable decrease in the density of the mixture (Table 2). However, at the same time, this implies that there is a greater surface of cork in the mixture which may prevent the correct cohesion of the TPU, and that the printing defects are obtained to the point where it is not suitable to introduce more cork into the mix.

The result of the melt flow index (MFI) for raw TPU was 54.0 ± 13.9 g/10 min, aligned with the manufacturer data sheet. The measured MFI values of TPU + 1 wt% cork and TPU + 3 wt% cork are 61.9 ± 5.6 g/10 min and 39.2 ± 4.4 g/10 min, respectively. This material presents a remarkable fluidity and, as could be guessed, the MFI decreases as the amount of filler increases. This does not happen to the samples loaded with cork at 1%wt due to the high dispersion of the cork powder; this dispersion may be due to the low amount of filler to be dispersed in the matrix, thus allowing the filler mobility; however, in any case, the material is still in the range of processability required by FGF processes.

**Figure 3:**
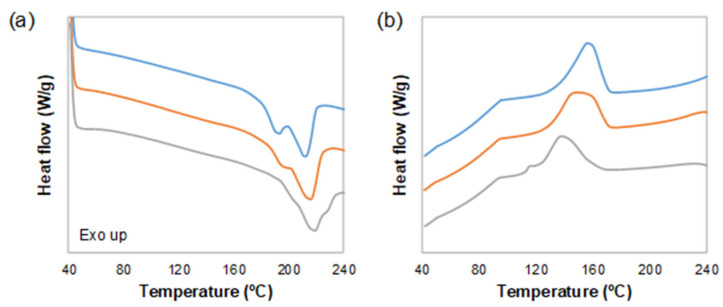
DSC thermograms of TPU (blue), TPU + 1 wt% cork (orange) and TPU + 3 wt% cork (grey); (**a**,**b**) represent the heating cycle and the cooling cycle, respectively.

The thermal properties of the cork composites were analysed via DSC and TGA. Figure 3 shows the DSC thermograms of the different materials prepared in this study when heated and cooled under a nitrogen atmosphere. It can be observed that the TPU melting point (local minimum in the endotherm peak in Figure 3a) increases when the amount of cork is higher. Moreover, the cold crystallization temperature of TPU (local maximum in the exothermic peak at 120–160 °C in Figure 3b) decreases the presence of cork. The degradation of cork fillers whilst being extruded should be taken into account, as normally, the degradation occurs between 200 °C and 250 °C. Therefore, a low melting temperature is a must for the matrix selection [14]. The cold crystallization enthalpies obtained were 21.8, 20.3 and 17.1 J/g for TPU, TPU + 1 wt% cork and TPU + 3 wt% cork, respectively. This could mean that the cork particles are able to delay the crystallization of the TPU domains, but also reduce the energy necessary to carry out this process. When these enthalpies are compared to the enthalpy of fusion, similar values are obtained, implying that cork does not impede the recrystallization of TPU.

**Figure 4:**
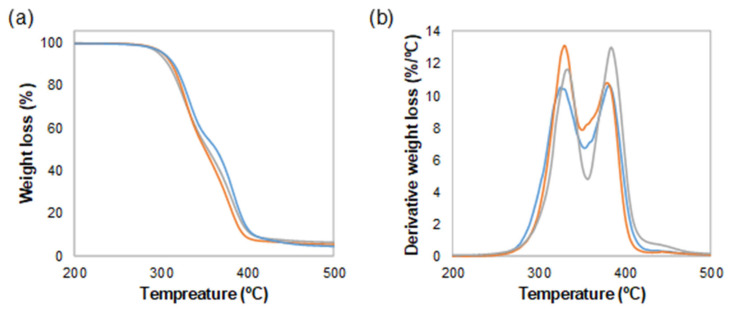
(**a**) Weight loss and (**b**) derivative weight loss thermograms of TPU (blue), TPU + 1 wt% cork (orange) and TPU + 3 wt% cork (grey).

The TGA analysis presented in Figure 4 shows that the cork particles may have a small effect in increasing the onset temperature of TPU. TPU does not have a particularly high degradation temperature [29], so cork may confer the composite a certain increase in the thermal stability, even though it is an organic filler. This effect is more clearly observed in the derivative weight loss curves in Figure 4b. The local maximum temperatures, corresponding to the maximum degradation rates of the material, are 327, 329 and 335 °C for the first peak and 376, 382 and 385 °C for the second peak, for TPU, TPU + 1 wt% cork and TPU + 3 wt% cork, respectively. Hence, even though the amounts of cork used are rather low in these thermoplastic composites, a certain increase in the thermal stability can be realized.

According to the DSC and MFI results, it can be stated that the TPU cork blends can be successfully processed within a temperature range between 160 °C and 200 °C.

**Figure 5:**
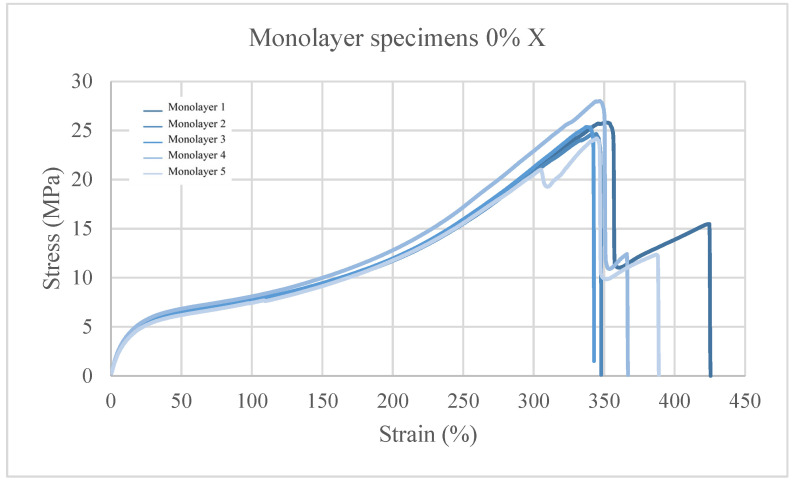

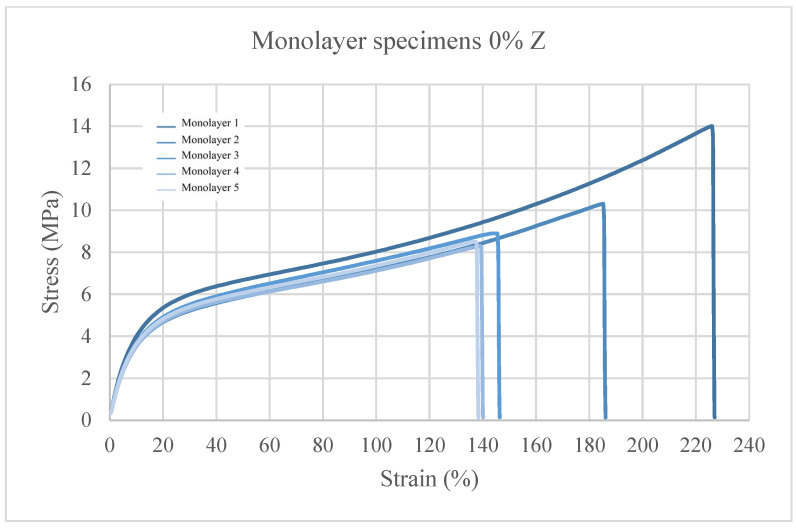
Stress–strain graphs for TPU + 0 wt% C.

In the study of the stress–strain graphs (Figure 5) of the neat TPU specimens, it is observed that all the X-oriented specimens break at a similar stress and at a similar deformation, where there are also jumps in the measurements. This phenomenon occurs because, as the specimen stretches, the chords are broken separately, generating delamination and a discontinuous fracture. In the Z orientation, a fracture is observed at the interfaces of the deposited filament, this being the reason for the lower values of both the maximum stress and deformation compared to x orientation.

**Figure 6:**
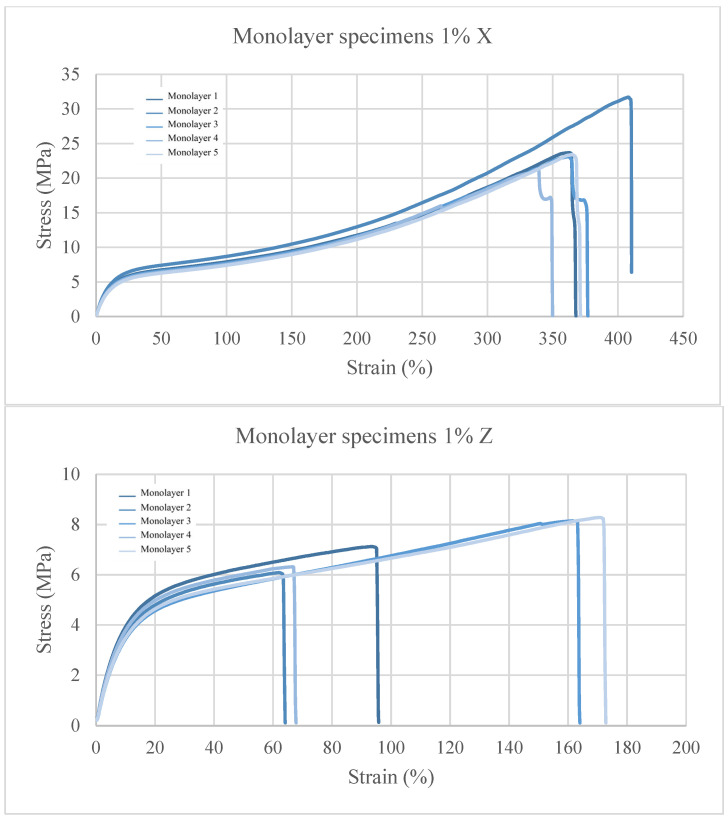
Stress–strain graphs for TPU + 1 wt% C.

Regarding the specimens with 1 wt.% cork shown in Figure 6, a decrease with respect to the virgin TPU printed in the x orientation of 6.29% in the maximum stress and an increase of 6.69% in the maximum deformation is observed. With regard to the specimens with orientation in Z, they have a decrease in both maximum stress and maximum deformation of 28.1% and 33.73%, respectively, with respect to virgin TPU.

In these graphs, a greater variation in values among specimens is observed, but a difference in the elastic and plastic behaviour is not realized because the slopes are equal. Only a lower tensile strength is observed, which may be caused by the inclusion of solid particles which generate defects during the fabrication process and cause bubbles and a lack of adhesion in the material. This is likely to lead to a possible lack of cohesion in the material because the cork has not been mixed uniformly with TPU.

**Figure 7:**
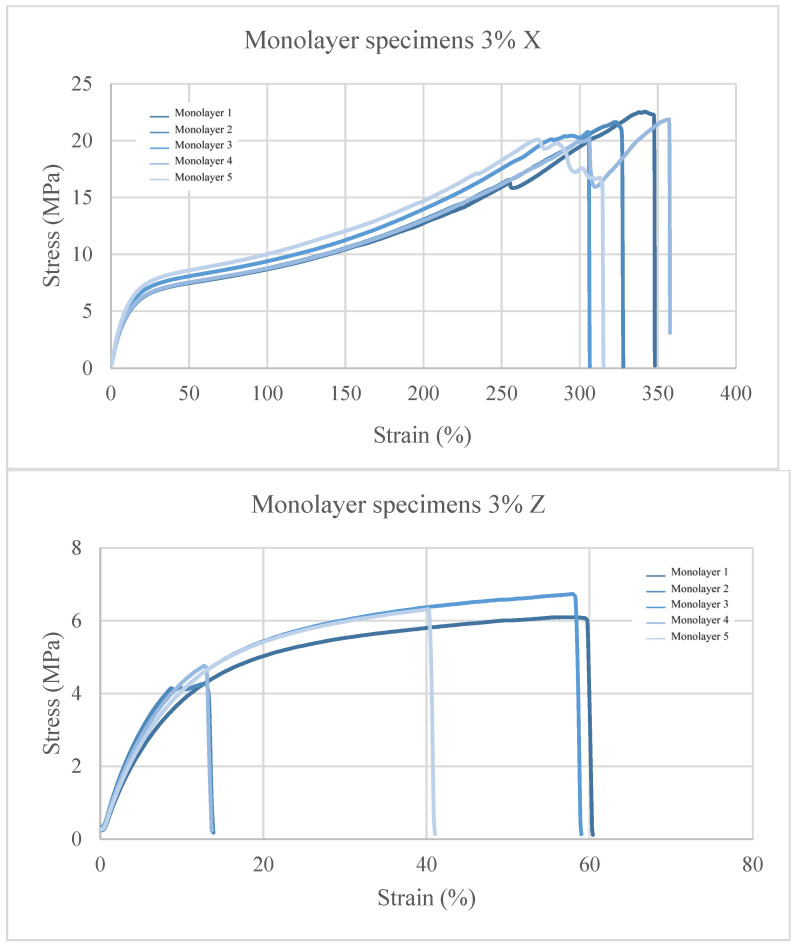
Stress–strain graphs for TPU + 3 wt% C.

In the tests at 3% cork content (Figure 7), the X-oriented samples show a reduction in the maximum stress of 14% and a decrease in the maximum strain of 7.27% with respect to the virgin TPU. In the Z orientation, the composites show a decrease of 50% and 78.92% in maximum stress and strain, respectively. This shows a trend where the mechanical properties decrease when the cork filler content is increased in the mixture. This is likely due to the lack of compatibility between the TPU matrix (hydrophobic) and the cork particles (highly hydrophilic) [20]. This may favour the aggregation of cork particles originating from a macrophase separation, which can be the origin of potential fracture points, causing the failure of the material at a lower stress level than that in the case of virgin TPU.

Table 3 summarizes the mechanical properties obtained from tensile testing. The decrease in both the maximum strength and maximum strain is not too high in the TPU–cork composites printed in the X direction. In particular, the TPU + 1 wt% C does not present significant differences in these mechanical parameters when compared to virgin TPU. In the Z-oriented specimens, a more significant decrease is observed in these values, suggesting that cork particles worsen the adhesion between layers, due to the lack of compatibility with the TPU matrix, as already explained. The Young’s modulus, however, increases significantly with the cork content in all the cases studied. This indicates that cork increases the stiffness of the material without significantly compromising their ductility, at least when the specimens are printed in the x direction. These results are in good agreement with previous research, where a reduction in mechanical properties was also observed [13,18,20,30,31].

**Figure 8:**
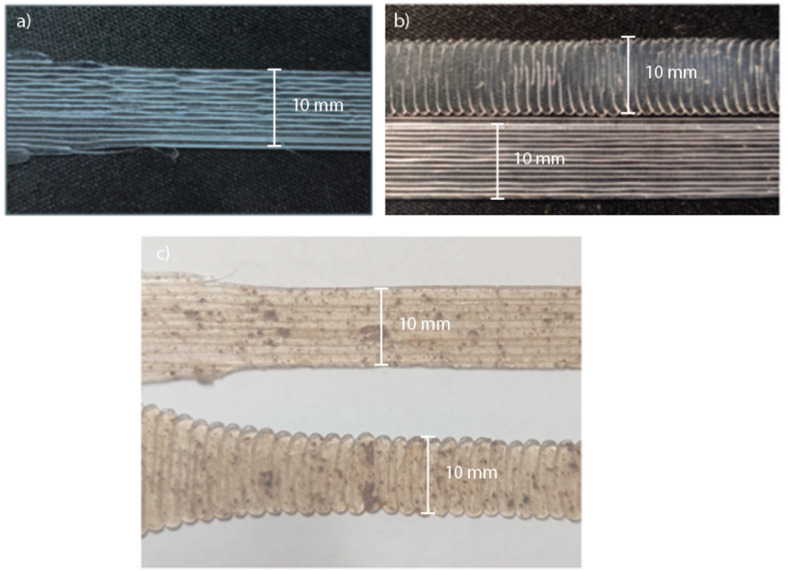
(**a**) Layer defects and (**b**) different printing orientations of non-additivated TPU. (**c**) TPU + 3 wt% cork inclusions in the tensile specimens.

Figure 5, Figure 6 and Figure 7 show a greater deviation in the breaking point of the specimens when the cork content is increased. As shown in Figure 8, the cork particles tend to aggregate causing more defects due to the low homogeneity of the mixture together with the defects caused in the printing. This can be particularly clearly observed in TPU + 3 wt% C specimens, which breaks in regions where the accumulation of cork particles is higher. Moreover, typical defects in AM, like bubbles, variations in the printing flow or low layer adhesion caused by material clogging because of the cork particles, also contribute to decrease the ductility of the material.

Nozzle clogging can be avoided with a correct selection of the nozzle diameter; in this case, a 0.8 mm was selected, which implies a safety factor of 4 with regard to the biggest particle size. In any case, it must be considered that the increase in the nozzle size demands a larger extrusion system. This means that the larger the nozzle is, the greater of the larger the additives incorporated to the polymeric matrix can be (i.e., it is widely used for short fibre-reinforced polymers), thus requiring longer extrusion screws that will melt the polymers up to an optimal fluent state to obtain a constant and homogeneous flow.

Also, when scaling this process, twin-screw extrusion systems are recommended to obtain the composite to improve the level of dispersion of the blends. This fact, together the elastic nature of TPU, increases the difficulty of the blends due to its extension and compression behaviour that can block the movement of the particles., This dispersion generates different filler concentrations in the specimens and, therefore, indicates the variability of the results.

Table 4 shows the coefficient of variation (CV), calculated as the ratio between the mean and standard deviation (used in this work to measure the errors of the mechanical parameters), and indicates the repeatability of the measurements. It can be observed that the CV of the Z samples is, in general, higher than that of the X samples, indicating that the specimens printed in this orientation present lower repeatability, especially in the case of the maximum strain. However, it is generally accepted that the measurements of the maximum strain (also known as elongation at break) have a high dispersion when elastic materials such as TPU are used, as we previously observed [32]. In any case, in general, all the CV values are below 10%, indicating that the repeatability of these measurements is acceptable.

The anisotropy in the mechanical properties observed between the X and Z orientations, which is due to the higher number of interlayer bonds between trajectories in the z-oriented specimens, causes more potential defects facilitating that the material breaks at lower elongations. This effect is characteristic of extrusion-based AM technologies [32].

**Figure 9:**
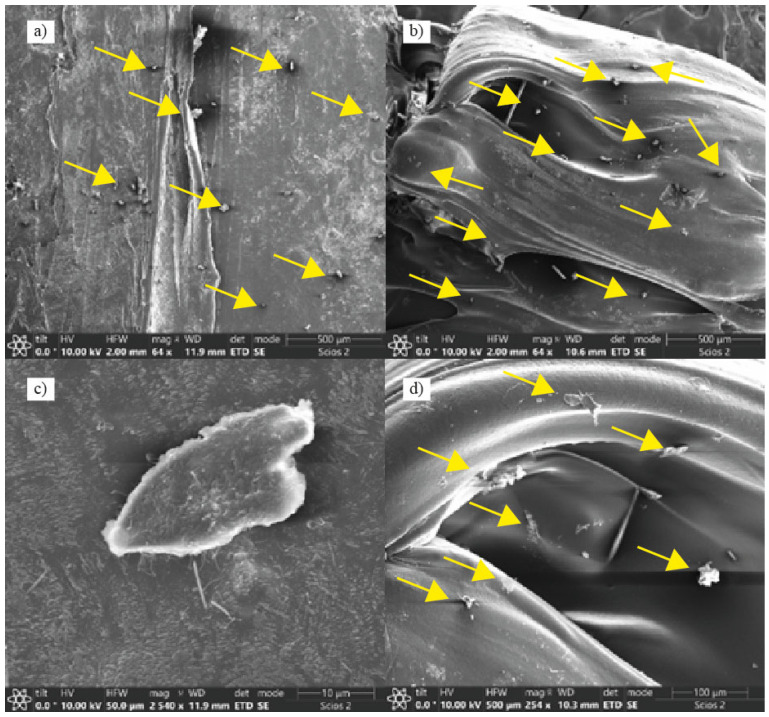
SEM micrographs of TPU + 1 wt% C pellets. Yellow arrows indicate position of the cork particles within the TPU matrix; (**a**,**b**) reveal the cork particles dispersion in the TPU matrix; (**c**) presents a single cork powder particles integrated in the matrix; and (**d**) presents the dispersion of the cork powder next to a void.

SEM microscopy analysis has been performed to understand the integration and dispersion of the cork particles in the TPU matrix. The study reveals that the particles are not uniformly dispersed, finding local agglomerations with an unstructured layout, as the yellow arrows reveal in Figure 9. Particles tend to be in the surface of the extruded filaments, and there is not a good integration within the polymeric matrix, also as shown in Figure 9. This fact confirms that the decrease in the mechanical process is due to the lack of integration and suggests the need for integration agents when mixing natural fillers like cork in TPU matrixes.

## 4. Conclusions

In this research, the process of developing a polymeric material blend has been presented in detail, with the case study of a TPU blend with cork particles at different concentrations for use in fused granular fabrication processes. The limits for blend mixes have been explored by defining 3% in weight of filler as the limit according to the equipment used.

The MFI, DSC and TGA results define the temperature ranges where these material blends can be successfully processed. Thermal characterization has been performed by defining the operative process temperatures between 160 °C and 200 °C with an MFI that facilitates the processing of the blends via FGF. Taking this into account, monolayer specimens have been printed with different orientations to obtain the mechanical properties of these materials when manufactured via FGF.

The results reveal a decrease in mechanical properties as the cork particles are incorporated into the TPU matrix. There is a 53.52% reduction in the maximum stress on the x-axis and an 81.82% decrease in the values obtained with specimens oriented on the z-axis. The maximum deformations have also been reduced by 37.3% on the x-axis and by 69.77% on the z-axis. These results are caused by the lack of compatibility between the cork particles and TPU, together with processing defects. The inclusions of these natural fillers also hinder the mobility of the polymeric chains as the material is deposited. Nevertheless, a significant weight reduction is realized as the density of the final blends is decreased when the filler is incorporated. The cork content is increased, reaching values in density of 0.96 g/cm^3^ for 3 wt% cork composites, leading to a weight reduction of 21.31% when compared to TPU. These results define the first steps in the search for applications of novel composites in industry. However, there are limitations of this study in terms of the amount of cork powder to be blended, or the complexity of being processed together with the scalability to industrial volumes. Despite this, the properties of the material described in this study suggest its potential use in applications where the produced parts require significant weight reductions, as could be the case in packaging industries, where the incorporation of cork particles will reduce the industry carbon footprint by using more natural fillers obtained from industrial waste and less petrol-based derivatives.

## Figures and Tables

**Table 1:** Printing parameters of the TPU-Cork monolayer specimens.

Nozzle diameter	0.8 mm
Flow rate in slicer	0.6
Flow rate in slicer for blends	1.3
Layer height	0.6 mm
Infill density	100%
Perimeters	0
Retractions	Not activated
Infill pattern	Rectilinear
Print speed	60 mm/s
Nozzle temperature	230 °C
Bed temperature	40 °C

**Table 2:** Mass percentage, volume and theoretical density of the blends.

	TPU	TPU + 1 wt% C.	TPU + 3 wt% C.
Cork amount (wt%)	0	1	3
Cork amount (vol%)	0	9.3	23.9
Density (g/cm^3^)	1.22	1.12	0.96

**Table 3:** Mechanical properties of the materials printed in the X and Z orientations of TPU (0%), TPU + 1 wt% C (1%) and TPU + 3 wt% C (3%).

	0% X	0% Z	1% X	1% Z	3% X	3% Z
Young’s Modulus (MPa)	50.0 ± 2.7	49.5 ± 2.8	53.3 ± 3.4	50.0 ± 3.1	66.2 ± 4.8	57.5 ± 4.7
Maximum Stress (MPa)	25.6 ± 1.5	10.0 ± 2.4	24.66 ± 4.1	7.1 ± 1	21.3 ± 1	5.6 ± 1.1
Maximum Strain (%)	344.8 ± 4.9	166.2 ± 38.6	367.1 ± 25.3	110.7 ± 25	319.9 ± 32.5	35.9 ± 22.3

**Table 4:** Coefficient of variation (CV) of the materials printed in the X and Z orientations of TPU (0%), TPU + 1 wt% C (1%) and TPU + 3 wt% C (3%).

	0% X	0% Z	1% X	1% Z	3% X	3% Z
Young’s Modulus (MPa)	5.4%	5.65%	6.37%	6.2%	7.25%	8.21%
Maximum Stress (MPa)	5.85%	24%	16.62%	14.08%	4.7%	19.64%
Maximum Strain (%)	1.42%	23.22%	6.89%	22.58%	10.15%	63.78%

## Data Availability

The data presented in this study are available on request from the corresponding author. The data are not publicly available due to privacy.
